# Delineation of Stage Specific Expression of *Plasmodium falciparum* EBA-175 by Biologically Functional Region II Monoclonal Antibodies

**DOI:** 10.1371/journal.pone.0018393

**Published:** 2011-04-14

**Authors:** B. Kim Lee Sim, David L. Narum, Rana Chattopadhyay, Adriana Ahumada, J. David Haynes, Steven R. Fuhrmann, Jennifer N. Wingard, Hong Liang, J. Kathleen Moch, Stephen L. Hoffman

**Affiliations:** 1 Protein Potential LLC, Rockville, Maryland, United States of America; 2 Laboratory of Malaria Immunology and Vaccinology, National Institute of Allergy and Infectious Diseases, National Institutes of Health, Rockville, Maryland, United States of America; 3 Sanaria Inc., Rockville, Maryland, United States of America; 4 U.S. Military Malaria Vaccine Program, Silver Spring, Maryland, United States of America; 5 Intercell USA, Gaithersburg, Maryland, United States of America; 6 Department of Molecular Physiology and Biophysics, University of Virginia, Charlottesville, Virginia, United States of America; 7 Infectious Disease Research Institute, Seattle, Washington, United States of America; New York University School of Medicine, United States of America

## Abstract

**Background:**

The malaria parasite *Plasmodium falciparum* EBA-175 binds its receptor sialic acids on glycophorin A when invading erythrocytes. The receptor-binding region (RII) contains two cysteine-rich domains with similar cysteine motifs (F1 and F2). Functional relationships between F1 and F2 domains and characterization of EBA-175 were studied using specific monoclonal antibodies (mAbs) against these domains.

**Methods and Findings:**

Five mAbs specific for F1 or F2 were generated. Three mAbs specific for F2 potently blocked binding of EBA-175 to erythrocytes, and merozoite invasion of erythrocytes (IC_50_ 10 to 100 µg/ml IgG in growth inhibition assays). A mAb specific for F1 blocked EBA-175 binding and merozoite invasion less effectively. The difference observed between the IC_50_ of F1 and F2 mAbs was not due to differing association and disassociation rates as determined by surface plasmon resonance. Four of the mAbs recognized conformation-dependent epitopes within F1 or F2. Used in combination, F1 and F2 mAbs blocked the binding of native EBA-175 to erythrocytes and inhibited parasite invasion synergistically *in vitro*. MAb R217, the most potent, did not recognize sporozoites, 3-day hepatocyte stage parasites, nor rings, trophozoites, gametocytes, retorts, ookinetes, and oocysts but recognized 6-day hepatocyte stage parasites, and schizonts. Even though efficient at blocking binding to erythrocytes and inhibiting invasion into erythrocytes, MAb R217 did not inhibit sporozoite invasion and development in hepatocytes *in vitro*.

**Conclusions:**

The role of the F1 and F2 domains in erythrocyte invasion and binding was elucidated with mAbs. These mAbs interfere with native EBA-175 binding to erythrocyte in a synergistic fashion. The stage specific expression of EBA-175 showed that the primary focus of activity was the merozoite stage. A recombinant RII protein vaccine consisting of both F1 and F2 domains that could induce synergistic activity should be optimal for induction of antibody responses that interfere with merozoite invasion of erythrocytes.

## Introduction

The invasion of erythrocytes by malaria parasites is mediated by specific molecular interactions between erythrocyte receptors and parasite ligands. Within *Plasmodium* sp., a family of erythrocyte binding proteins (EBPs) with signature cysteine rich motifs is involved in binding to erythrocyte receptors for invasion of erythrocytes [1]–[4] or sequestration of parasitized erythrocytes to endothelial cells [5]–[7]. EBA-175 is a 175 kDa erythrocyte binding protein [1], [4], [8], [9], that binds erythrocytes via sialic acids on its receptor glycophorin A. This binding involves recognition of both the sialic acids and the peptide backbone of glycophorin A [4]. The erythrocyte-binding region of EBA-175 is a 616 amino acid fragment, designated region II (RII) that contains 27 cysteines as tandem duplications of two copies of a cysteine rich domain to form regions F1 and F2 [2]. F1 and F2 are homologous to the Duffy binding protein of *P. vivax* and are also called Duffy binding-like domains (DBL). The presence of one or two DBL domains and other elements including a C-terminal cysteine-rich region and a type I transmembrane domain classifies EBA-175 as a member of the erythrocyte binding-like (EBL) superfamily of proteins [2]. This EBL superfamily includes BAEBL/EBA-140/EBP2 [10]-[12] MAEBL [13], EBA-181/JESEBL [14]and *var* genes, a large family of genes regulated by chromatin modification [15], [16] that encode antigenically variant proteins including PfEMP-1 [5], [7]. PfEMP-1 contains several DBL domains and is involved in the cytoadherence of parasitized erythrocytes to endothelia of microcapillaries causing cerebral malaria and mortality associated with *P. falciparum*. Thus the RII of EBA-175 that consists of two DBL domains that appear unique to *Plasmodium sp*. may constitute a good target for rational drug design and vaccines.

Antibodies generated against EBA-175 RII potently block the binding of native EBA-175 to erythrocytes [17]–[19] and even inhibit invasion of *P. falciparum* strains that invade erythrocytes by pathways that do not require sialic acids for invasion *in vitro* [18]. Further, a DNA prime/recombinant protein boost immunization regimen was shown to protect 3 of 4 *Aotus* monkeys against a lethal *P. falciparum* blood-stage challenge [17]. Immunoglobulin G obtained from the vaccinated *Aotus* monkeys inhibited parasite growth *in vitro* [17]. F2 alone is sufficient for binding to erythrocytes [4]. Indeed, crystallographic studies show that binding to glycophorin A occurs when a dimer arrangement of RII forms a channel allowing ligand-receptor interaction wherein more than 75% of the sequences within the channel are derived from F2 [20]. The EBA-175 RII DNA vaccine tested in non-human primates [17], [19] and recombinant protein vaccine tested in a Phase 1 clinical trial [21] are comprised of both F1 and F2 domains.

In this report we investigated the role of EBA-175 RII F1 and F2 domains in erythrocyte binding and parasite invasion *in vitro* using monoclonal antibodies (mAbs) that specifically recognized F1 or F2 domains, and established the stage specific expression of EBA-175 in the parasite life cycle. Although RII specific mAbs blocked F2 function more efficiently than F1, we show that a combination of F1 and F2 specific mAbs blocked the function of EBA-175 erythrocyte binding and parasite invasion *in vitro*, synergistically.

## Results

### Characteristics of MAbs generated

**Figure 1:**
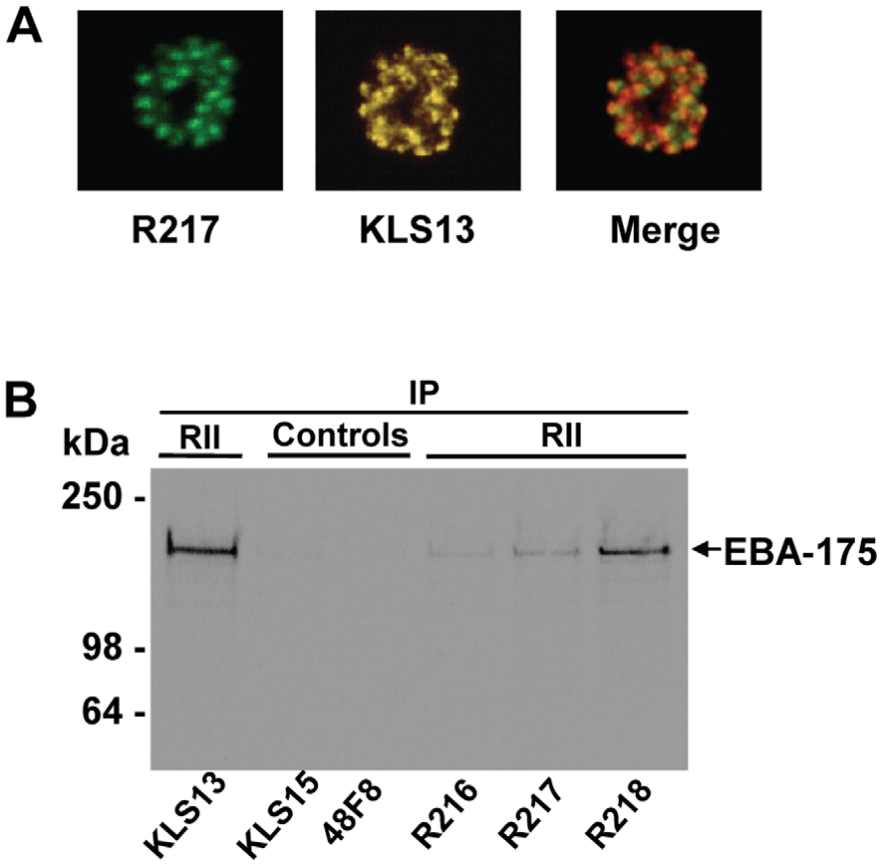
EBA-175 RII mAbs generated against baculovirus expressed recombinant EBA-175 RII protein recognizes native EBA-175. Panel A: Dual immunofluorescent analyses showing apical staining of mature *P. falciparum* (FVO strain) schizont with EBA-175 RII specific mAb R217 used at 10 ug/mL and rabbit polyclonal sera KLS13 against baculovirus expressed EBA-175 RII (used at 1:200 dilution). Panel B: Phosphoimager detection of parasite culture supernatant containing [35S]-labeled native EBA-175 immunoprecipitated with mAbs and polyclonal sera. MAb R216, R217, R218 and KLS13 (polyclonal sera against EBA-175 RII) immunoprecipitated native EBA-175, whereas mAb 48F8 (isotype control) and polyclonal sera KLS15 raised against Freund’s adjuvant did not.

Five mAbs were cloned and identified as R215 through R218 and R256. All the mAbs were identified as belonging to subclass IgG1. These mAbs gave a characteristic apical punctate fluorescence pattern with FVO (Fig. 1A and Table 1) and 3D7 strain (not shown) late stage schizonts. The specificity of the mAbs against EBA-175 was determined by immunoprecipitation of *P. falciparum* [^35^S]-metabolically labeled schizont stage parasite culture supernatant containing labeled native EBA-175 (Fig. 1B) as well as schizont-infected erythrocyte lysates (not shown). MAbs R216, R217 and R218 recognized a 175 kDa protein, R216 albeit poorly, that was similar in mass to EBA-175 as recognized by rabbit polyclonal anti-EBA-175 RII (KLS13) used as a positive control (Fig. 1B) [18], and negative control KLS15, rabbit polyclonal sera raised against adjuvant alone. The isotype control mAb 48F8, a mAb raised against adjuvant alone did not immunoprecipitate EBA-175 (Fig. 1B). The IFA staining patterns of mAbs R215, R216, R218 and R256 and results of immunoprecipitation with mAbs R215 and R256 were similar to that with mAb R217, respectively (data not shown).

**Table 1:** Summary of EBA-175 RII specific mAbs.

mAb	Isotype	IFA(Apical Staining)	Epitope mapping				KD of binding (M)	IC_50_-blocking EBA-175 binding (ng/ml)	IC_50_-GIA#
			RII domain	ImmpptEBA-175	Immunoblotting				
					Reduced	Non- reduced			
R215	IgG1	+	F2c^1^	+	-	+	ND	ND	ND
R216	IgG1	+	F2L	Poor	+	-**	ND	NA	NA
R217	IgG1	+	F2c^1^	+	-	+	1.84×10^−9^	1–15	10–100 µg/ml
R218	IgG1	+	F1c	+	-*	+	1.53×10^−11^	25–125	>1 mg/ml
R256	IgG1	+	F2c^1^	+	-	+	1.8×10^−10^	1–15	10–100 µg/ml

ND: not determined

NA: not achievable. R216 at 333 and 666 µg/ml IgG blocked EBA-175 binding by 14 and 21%, respectively.

# *P. falciparum* FVO 2 cycle suspension growth inhibition assay (GIA).

1 mAbs compete against each other for binding RII by competition ELISA.

c: constrained epitope; L: linear epitope; immppt: immunoprecipitate.

*incomplete reduction; ** partial denaturation/reduction.

**Figure 2:**
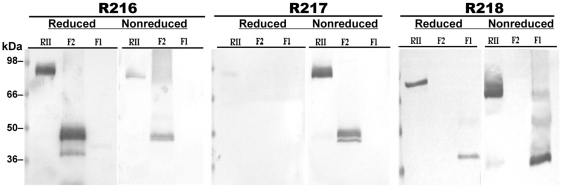
Immunoblot analysis of EBA-175 RII mAbs against *P. pastoris* expressed recombinant RII F1 or F2 domains. MAb R216 recognized a linear epitope within the F2 domain reacting against both reduced recombinant RII and F2 domain. MAb R217 recognized an epitope within F2 that was conformationally dependent. Reduction abrogated reactivity of R217 against recombinant RII and the F2 domain. MAb R218 was conformationally dependent and specific against the F1 domain and reacted against non-reduced RII and F1. Purified recombinant baculovirus EBA-175 RII protein at 0.5 µg per lane, or 10 uL per lane of supernatant of *P. pastoris* cultures expressing recombinant EBA-175 RII F1 or F2 domains were separated by SDS-PAGE under reduced or non-reduced conditions and electroblotted onto nitrocellulose membranes. In analyses against R216, a small fraction of the recombinant proteins were slightly denatured or reduced. In analysis using R218, reduction of the recombinant proteins was not absolute. Membranes were probed with 10 ug/mL each of mAbs R216, R217 or R218 separately. A similar staining pattern to that of R217 was observed for mAbs R215 and R256 (data not shown).

Immunoblot analysis using both reduced and non-reduced conditions showed that mAbs R217 (Fig. 2), R215 and R256 (data not shown) recognized a conformational, disulfide-constrained epitope located within the F2 domain of RII. This is because R217 only recognized non-reduced F2 domain of RII. MAb R218 recognized a conformation-dependent epitope located within the F1 domain of RII because only the F1 domain was recognized. Even though similar quantities of F1 and F2 protein were present in the immunoblot, the recognition was much stronger against non-reduced F1, probably because reduction was incomplete. MAb R216 most probably recognized a linear or unexposed region of the F2 domain of RII because only F2 was recognized. Even though similar quantities of F2 protein were present in the immunoblot, the recognition was much stronger against reduced F2 (Fig. 2). Slight recognition of non-reduced F2 was seen with R216 probably because there was some denaturation and reduction in the material. Further analysis of RII/F2 specific mAbs R215, R216, R217 and R256 demonstrated that R215, R217 and R256 competed with each other for binding recombinant RII protein by competition ELISA (data not shown).

### MAbs generated blocked the binding of native EBA-175 to erythrocytes and inhibit invasion and growth of parasites *in vitro*

**Figure 3:**
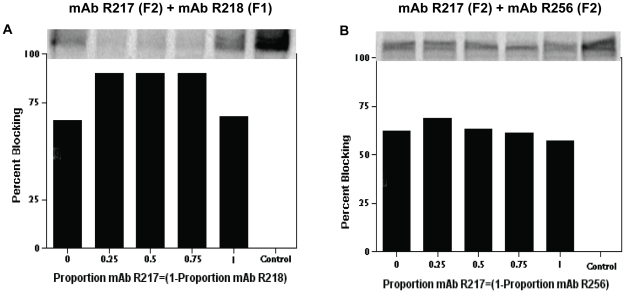
MAbs against the F1 and F2 domains block native [^35^S]-labeled EBA-175 binding to erythrocytes synergistically. Effects of mAbs on immunoprecipitation of [^35^S]-labeled parasite culture supernatant containing labeled native EBA-175. Panel A shows that ratios of mAb R217 (against F2) and R218 (against F1) together increased the blocking of native EBA-175 binding (a synergistic effect). In contrast, Panel B shows that different ratios of mAb R217 (against F2) and R256 (also against F2) together resulted in similar levels of blocking (an additive effect). R217 and R256 may recognize a common epitope within the F2 domain. Bars show % blocking values as assessed by a phosphoimager.

Differences in the ability of recombinant F1 and F2 domains to bind erythrocytes have been reported [4], [22]. When expressed on the surface of COS cells, F2 alone could support the binding and rosetting of erythrocytes on the surface of these COS cells, but not F1 alone [4]. Hence we investigated whether targeting F1 and F2 in combination would have an additive or synergistic effect on blocking of EBA-175 erythrocyte binding. Using IgG concentrations within the range of the IC_50_’s for R217, R218 and R256 (Table 1), and [^35^S]-metabolically labeled native EBA-175, blocking of binding studies were performed such that the proportion of mAb R217 equaled one minus the proportion of mAb R218 or R256. MAbs R217 and R256, both against the F2 domain, blocked [^35^S]-labeled native EBA-175 binding to erythrocytes efficiently with IC_50_ of 1-15 ng/mL IgG (Fig. 3 and Table 1). The MAb R218 against the F1 domain had a lesser ability to block the binding of native EBA-175 to erythrocytes (IC_50_ ranged between 25 to 125 ng/mL IgG, (Fig. 3 and Table 1). Interestingly, MAb R216 that recognizes a linear or unexposed epitope essentially could not block binding of native EBA-175 to erythrocytes at the maximal amount of MAb R216 used in our binding assay. In fact, the IC_50_ was only 14% and 21% at 333 and 666 µg/mL R216 (data not shown). When the F2 specific mAb R217 is proportionally mixed with the F1 specific mAb R218, a synergistic increase in blocking is evident (Fig. 3, panel A). Under similar conditions, proportional ratios of the mAb R217 mixed with mAb R256 both of which are most probably against a common region in F2 together resulted in an additive effect such that there was no overall increase in blocking (Fig. 3, panel B).

**Figure 4:**
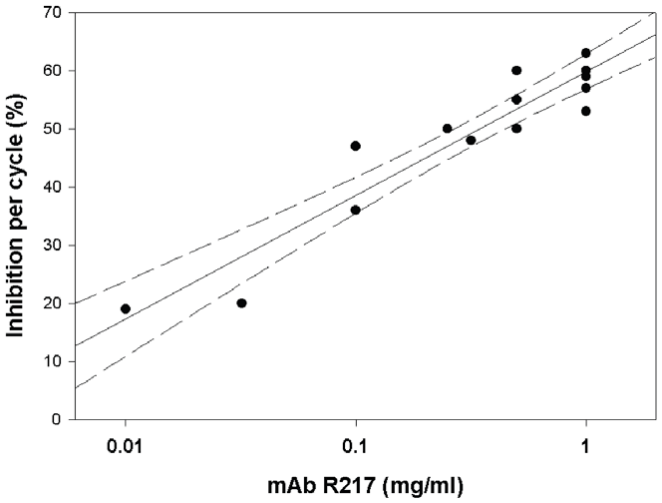
Scatterplot showing a dose effect on *P. falciparum* FVO growth using mAb R217. The solid line shows the linear regression and the dashed lines represent 95% confidence intervals. The goodness of fit R^2^ was 0.9017.

We investigated whether RII specific mAbs conferred growth inhibitory activity (GIA) on the parasite strains 3D7 and FVO. We selected the 3D7 strain that invades neuraminidase-treated erythrocytes, which are devoid of sialic acids, at 70% efficiency as compared to invasion of normal erythrocytes [23], and the FVO strain that is dependent on sialic acids for erythrocyte invasion [18]. The results of 5 separate growth inhibition assays are shown in Table 2 for mAbs R216, R217 and R218 and summarized for the FVO strain in Table 1. GIA results obtained for mAb R256 (data not shown) were similar to mAb R217. A dose dependent effect on parasite growth was observed with mAb R217 against the FVO strain (Fig. 4). The inhibition of parasite growth per cycle of the FVO strain compared to the 3D7 strain was approximately 2-fold greater. The IC_50_ for mAbs R217 and R256 (data not shown) against FVO ranged between 10 – 100 µg/mL IgG compared to the IC_50_ against 3D7 which was greater than 1 mg/mL (Table 2). Analysis of experimental conditions such as initial parasitemias and growth rates were similar and thus could not result in the consistent differences in inhibition of parasite growth between FVO and 3D7 strains (data not shown). Additional growth inhibition studies using mAbs R216 and R218, which recognized a linear or hidden epitope within F2 or a conformation-dependent epitope within F1 respectively, demonstrated that both significantly inhibited parasite growth although markedly less than mAb R217 (Table 2).

**Table 2:** Inhibition of growth of *P. falciparum* FVO and 3D7 strains by RII MAbs.

Exp. ^#^	mAb (mg/ml)	FVO infected RBCs (%)			3D7 infected RBCs (%)		
		48F8 (Control)	R217	R216	48F8 (Control)	R217	R216
		Pc*	Pt (Ipc)**	Pt (Ipc)	Pc	Pt (Ipc)	Pt (Ipc)
**1**	1.0	1.64	0.28 (59)	1.53 (4)	5.43	3.29 (22)	3.97 (14)
**2**	1.0	0.56	0.11 (57)	0.43 (13)	4.16	2.11 (29)	3.09 (14)
**3**	1.0	2.26	0.36 (60)	ND	5.38	3.16 (23)	ND
	0.32	2.34	0.63 (48)	ND	5.13	3.21 (21)	ND
	0.10	2.14	0.87 (36)	ND	5.18	3.23 (21)	ND
	0.03	2.40	1.52 (20)	ND	5.41	4.14 (12)	ND
	0.01	2.51	1.67 (19)	ND	5.25	4.62 (6)	ND

		48F8 (Control)	R217	R218	48F8 (Control)	R217	R218
4	1.0	0.97	ND	0.65 (18)	3.15	ND	3.02 (2)
	0.5	1.06	0.22 (55)	0.75 (16)	3.10	ND	3.15 (−1)
5	1.0	0.26	0.04 (60)	0.19 (14)	2.94	ND	2.82 (2)
	0.5	0.27	0.07 (50)	0.17 (20)	2.96	ND	2.86 (2)
	0.25	0.24	0.06 (50)	0.22 (6)	2.99	ND	2.79 (3)

*Pc: final parasitemia after two cycles of invasion in presence of control mAb 48F8.

**Pt (Ipc): final parasitemia after two cycles of invasion in presence of test mAb (calculated % inhibition per invasion cycle).

The percentage inhibition per cycle was calculated according to the formula: Ipc  =  1 – (Pt/Pc)^½^.

### Association and dissociation rates of mAbs R217 and R218 are similar

Since F1 and F2 domains have different avidities for binding human erythrocytes when transiently expressed on the surface of COS cells [4], we were interested in determining whether these differences could be probed using R218 and R217 that were against domains F1 and F2 respectively. Using surface plasmon resonance, we determined that the association and dissociation rates for mAbs R217 and R218, were similar, ka 2.35×10^5^ M^−1^s^−1^ vs ka 4.47×10^5^ M^−1^s^−1^, and kd 4.32×10^−4^ s^−1^ and kd 6.82×10^−6^ s^−1^, respectively. The difference in the kds would favor a higher affinity for mAb R218 that was not observed in our other assays. The kds for mAbs R217, R218 and R256 which were in the nanomolar or picomolar range are reported in Table 1.

### *P. falciparum* sporozoites do not express EBA-175 (Fig. 5)

**Figure 5:**
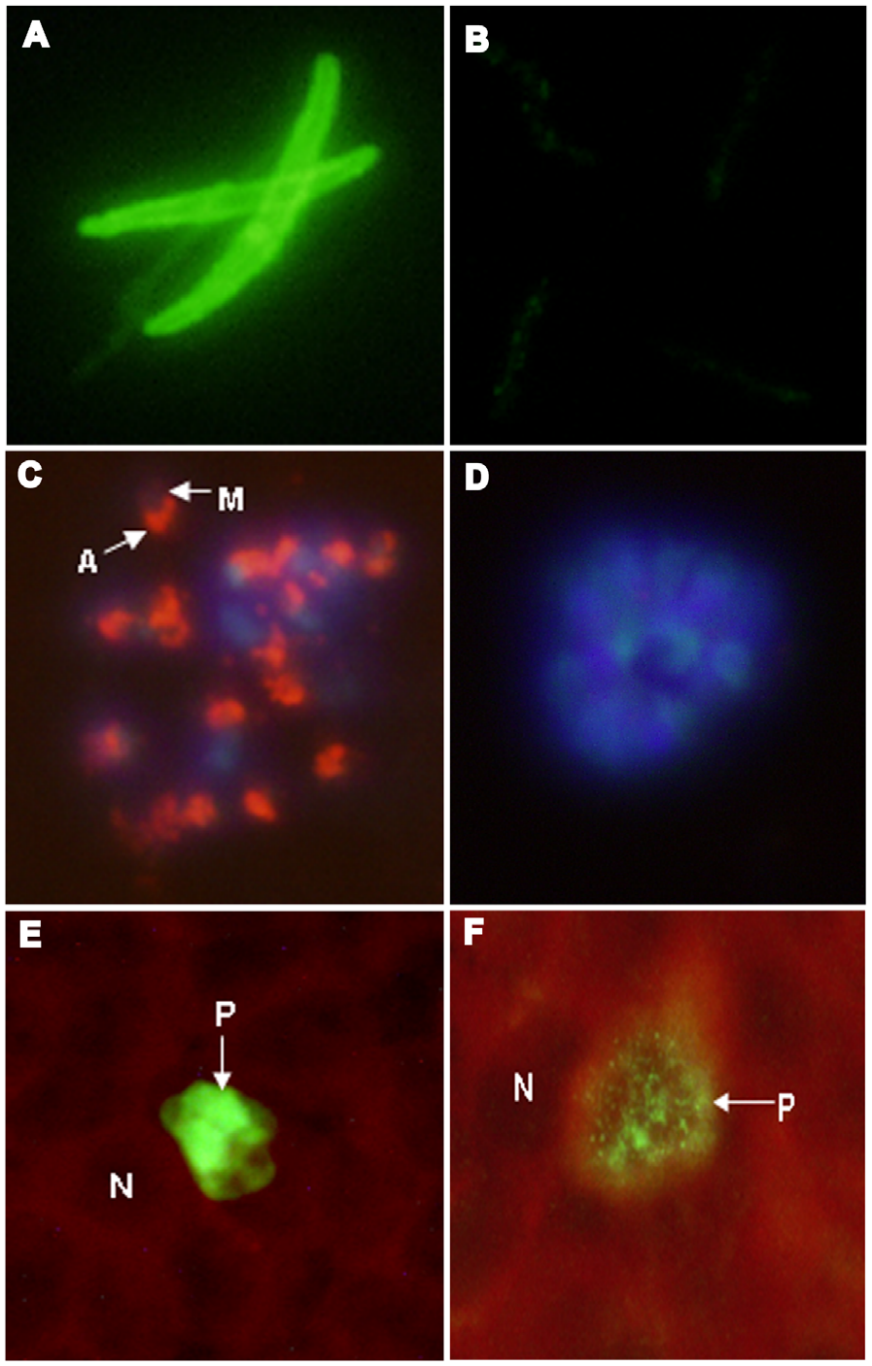
EBA-175 is expressed in *P.falciparum* schizonts, and late liver stages in human hepatocytes *in vitro*, but not sporozoites. A) Sporozoites stained with anti-PfCSP mAb 2A10 (used at 0.136 ug/mL), B) sporozoites stained with mAb R217 (used at 300 ug/mL), C) schizont stained with mAb R217 (used at 2.34 ug/mL), **M**: merozoite; **A**: apical end of the merozoite expressing EBA175, D) schizont stained with anti-PfCSP mAb 2A10 (used at 68 ug/mL). The nuclei were stained with DAPI, E) HC-O4 human hepatocytes stained with anti-*P.falciparum* liver stage antigen -1 (PfLSA-1) polyclonal rabbit serum (1:50 dilution) 6 days post infection with *P.falciparum* sporozoites, F) HC-O4 human hepatocytes stained with mAb R217 (used at 100 ug/mL) 6 days post infection with *P.falciparum* sporozoites. N: nucleus of the hepatocyte; P: liver stage parasite.

Immunofluorescence assays using mAb R217 against infectious *P. falciparum* sporozoites harvested from mosquito salivary glands and schizont infected human erythrocytes revealed that EBA-175 is expressed in mature schizonts (Fig. 1 and Fig. 5C), and as described previously [1], but not in sporozoites (Fig. 5B). R217 mAb failed to show any reactivity in IFA with sporozoites even when used at a concentration of 20 ug/mL (data not shown). In contrast, these sporozoites are recognized strongly by mAb 2A10, specific against *P. falciparum* circumsporozoite protein used at 0.136 ug/mL (Fig. 5A).

### EBA-175 is expressed in late hepatic stages (6 day) of P.falciparum, but not in early (3 day) hepatic stages in vitro (Fig. 5)

P. falciparum sporozoites when allowed to invade human HCO4 cells [24] in vitro, developed to 6 day liver stage parasites that express EBA-175 (Fig. 5F) . At 6 days after invasion of sporozoites, these liver stage parasites also strongly express MSP-1 (data not shown). In contrast, the earlier liver stage parasites 3 days after invasion of sporozoites, neither express EBA-175 nor MSP-1. MAb R217 recognized late (6 day) liver stage parasites that are probably merozoites with typical punctate fluorescence (Fig. 5F) prior to erythrocyte rupture, but did not recognize early (3 Day) hepatic stage parasites (Table 3).

**Table 3:** Expression of *P. falciparum* EBA-175 in late (6 day) but not in early (3 day) liver stages.

Sporozoites/well	Primary Antibody*	Days in culture	Number of liver stages			Mean (liver stages)/well	STDEV	% CV
			Well 1	Well 2	Well 3			
25,000	PfLSA-1	3	309	342	326	325.67	16.50	5.07%
	R217	3	0	0	0	0.00	0.00	0.00%
50,000	PfLSA-1	6	32	57	41	43.33	12.66	29.22
	R217	6	18	26	12	18.67	7.02	37.63

*PfLSA-1 polyclonal rabbit antibody used at 1∶50 dilution and mAb R217 used at 100 ug/mL.

### MAb R217 does not effect the invasion and development of *P. falciparum* sporozoites in human hepatocytes *in vitro* as assessed by the inhibition of liver stage development assay (ILSDA) (Table 4)

**Table 4:** MAb R217 does not inhibit invasion and development of *P. falciparum* sporozoites in the human hepatocyte line, HC04 as assessed by ILSDA.

Test Material	Number of Parasites Expressing PfLSA-1*					% Inhibition (compared to Medium)
	Well 1	Well 2	Well 3	Mean	SD	
Medium (control)	309	342	320	325.7	16.50	NA
Anti-PfEBA-175 mAb R217 (100 ug/ml)	367	331	314	337.3	27.06	0
Anti-PfCSP mAb 2A10 (100 ug/ml)	18	30	22	23.3	6.11	92.83

*PfLSA-1 polyclonal rabbit antibody used at 1∶50 dilution.

In ILSDA, mAb 2A10 against the *P. falciparum* CSP reduced the numbers of parasites expressing PfLSA-1 by 92.83%. In the same assay mAb R217 had no effect on the number of parasites expressing PfLSA-1 (Table 4).

### MAb 217 recognizes schizonts but not rings, trophozoites nor male and female gametocytes, retorts, ookinetes and oocysts

MAb 217 recognizes segmenters and merozoites in mature schizonts (Fig. 1A and Fig. 5C) but not early rings that develop just after invasion (2–6 hours), nor rings and earlier trophozoites (data not shown). Male and female gametocytes of all stages of development (stages I-V), retorts, ookinetes and oocysts are also not recognized by mAb 217 but these preparations are all recognized by monoclonal antibody against Pfs25 (from MR4, data not shown)[25].

## Discussion

RII is a 616 amino acid fragment of EBA-175 that consists of two cysteine-rich Duffy binding-like (DBL) domains (F1 and F2) that are homologous to the RII of Duffy binding protein of *P. vivax*. EBA-175 RII is the receptor-binding domain that binds sialic acids on its receptor, glycophorin A, on erythrocytes during merozoite invasion. Polyclonal antibodies from mice, rabbits and Aotus monkeys immunized against RII block native EBA-175 binding to erythrocyte and inhibit parasite growth *in vitro* [1], [19]. Here we report on the characterization of a panel of EBA-175 RII specific mAbs that both alone or in combination block the binding of native EBA-175 to erythrocytes and inhibit *P. falciparum* growth *in vitro*.

Five mAb clones designated R215 through R218 and R256 were developed against a purified baculovirus expressed RII (3D7 strain) protein [22] that recognized either linear (R216) or conformationally dependent (disulfide constrained) epitopes within F1 (R218) or F2 (R215, R217 and R256) as shown by immunoprecipitation (Fig. 1B) and immunoblot (Fig. 2). All of the mAbs recognized *P. falciparum* FVO and 3D7 schizont infected erythrocytes in standard fixed immunofluorescence assays. These strains were selected for testing because 3D7 can invade via sialic acid independent pathway(s) while FVO strictly invade by binding to sialic acids. All but one of the mAbs (R216) effectively blocked native EBA-175 binding and inhibited parasite growth *in vitro* (Table 1). MAb R216 was incapable of blocking the binding of native EBA-175 to erythrocytes and was also not able to inhibit in GIA (Table 1). The difference seen between mAb R216 and the other biologically active mAbs is likely due to R216 recognizing a linear or hidden epitope. Reactivity of mAb R216 to reduced recombinant RII in immunoblots show increased recognition as compared to non-reduced recombinant RII (Fig. 2). MAb R218 that recognized the F1 domain inhibited parasite growth considerably less than the F2 domain specific mAbs. Surface plasmon resonance association and dissociation rates revealed nanomolar or greater KDs for mAbs R217, R218 and R256 indicative of high affinity binding (KDs: 0.01–1 nM) (Table 1). Thus the observed differences in inhibition of parasite growth by the mAbs were not due to differences in affinity. It is possible that mAb218, which recognizes the F1 domain, is less able to inhibit parasite invasion because its recognition epitope is a region of F1that is not involved intimately with receptor binding. Structural studies of RII showed that F1 and F2 domains are very similar, and that RII crystallizes as a dimer which interacts extensively with each other in an antiparallel fashion resembling a handshake [20]. The F1 domain of one monomer interacts directly with the F2 domain of the other monomer. The formation of the RII dimer creates two channels that span the dimer with 75% of the residues that comprise the channel surfaces contributed by the F2 domain. Thus F1 is not physically involved in the binding but stabilizes the RII dimer complex required for docking of EBA-175 as it interacts with its receptor glycophorin A, as described in crystallographic studies [20].

Invasion of erythrocytes by the FVO strain is dependent on the presence of sialic acids on glycophorin A while 3D7 can invade by alternate pathways other than sialic acids. In the case of 3D7, antibodies against RII could nevertheless still significantly block invasion of erythrocytes [18], [26]. In this report we show that the F2 specific mAb R217 clearly blocked the invasion of the FVO strain approximately 2-fold greater relative to the 3D7 strain (Table 2). The RII amino acid sequences of the FVO versus the 3D7 strains only differ by 5 out of the 616 amino acid residues [27]. We thus argue that the reduced inhibition of invasion is due to the ability of 3D7 parasites to invade erythrocytes via an alternate pathway that does not involve sialic acids. MAb R217, on the other hand blocked the invasion of FVO parasites efficiently since FVO parasites exclusively use sialic acids on glycophorin A for invasion.

EBA-175 is most efficiently harvested from spent culture media of ruptured schizonts [8]. The fact that EBA-175 was a type I membrane protein [1] localized in micronemes [28] that was not primarily exposed, was confounding. More recently, it was shown that PfROM4, a rhomboid protease, specifically cleaves EBA-175 upon merozoite invasion of erythrocytes a process essential for further development of the parasite [29]. It remains to be elucidated whether the docking of the EBA-175 dimer bound to glycophorin A [20] and the release of EBA-175 from the merozoite by PfROM4 is a stepwise or simultaneous event. Obviously, the fact that mAb 217 and the other RII mAbs so efficiently inhibit invasion and binding argues that the functional EBA-175 molecules are those in close contact at the erythrocyte surface. It remains to be explained why mAb R217 blocks the invasion of sialic acid independent strains [18] even though PfROM4 cleaves EBA-175 equally in both sialic acid dependent and independent strains [29].

EBA-175 is expressed in the micronemes of merozoites in schizonts [28] probably as early as nascent segmenting stages in both liver stage and asexual stage schizonts. It had previously been reported that EBA-175 is expressed in sporozoites, a conclusion from studies of reverse transcription of sporozoite total RNA as well as immunofluorescence of sporozoites using polyclonal antibody raised against a recombinant fragment of EBA-175 [30]. We show that mAb R217 does not recognize sporozoites by IFA (Fig. 5) and does not recognize parasites developing in infected hepatocytes 3 days after infection with *P. falciparum* sporozoites (Table 4), nor developing and mature male and female gametocytes (all stages 1-V), retorts, ookinetes and oocysts. However, EBA-175 is clearly expressed in parasites developing in hepatocytes 6 days after sporozoite infection, at a time when these liver stage schizonts are close to being ready to rupture (Fig. 5F, Table 3). In summary, we find no evidence of sporozoite or early liver stage expression of EBA-175 using our characterized mAbs specific against EBA-175 RII. Our studies use preparations of sporozoites that are aseptic and purified from mosquito salivary gland material produced in conditions that meet regulatory compliance for production of clinical material, and liver assays that are used for assessment of such sporozoites [31]. The sporozoites used are characterized in terms of purity and asepticity [31]. At this time, we do not have an explanation for the discrepancy between our results and those of Gruner et al [30].

The mAb reagents specific for EBA-175 RII described here if used together with EBA-175-GFP chimeric parasites [32] could be powerful tools that can be used to probe the role of EBA-175 in merozoite invasion of erythrocytes and may lead to the design of improved receptor interfering drugs and vaccines.

## Materials and Methods

### Ethics Statement

All animal studies were done in compliance with protocols approved by the Animal Care and Use Committee at EntreMed Inc., 9640 Medical Center Dr., Rockville MD 20850, under approved protocol ID No. ENT018.

### Parasites

*Plasmodium falciparum* 3D7 strain and FVO strain (Aotus adapted) were maintained as previously reported [33]. Schizonts were purified on Percoll density gradients [34]. *P. falciparum* FVO strain was metabolically labeled with TRAN^35^S-LABEL™ (ICN Radiochemicals, Irvine, CA) as previously described [4]. Cell pellets and supernatant were stored at −70°C for later use.

### Animals and immunizations

BALB/c mice were immunized subcutaneously with 50 µg each of purified baculovirus derived RII protein (3D7) [22] in Freund’s complete and incomplete adjuvant as suggested by the manufacturer (Sigma, St Louis, MO). Mice were immunized on days 0, 14, and 28 and bled on days, −1, 12, 26, and 40.

### Monoclonal Antibodies

MAbs were produced using spleen cells obtained from BALB/c mice immunized with purified baculovirus recombinant EBA-175 RII protein (3D7) [22], and fused with Sp2/0-Ag14 myeloma cells [35]. Hybridoma culture supernatants were screened by indirect immunofluorescence assay (IFA) against methanol-fixed parasitized (3D7) erythrocytes prepared on 15 well slides. Immunoglobulin G was detected using a goat anti-mouse IgG (γ) chain specific FITC coupled reagent (Kirkegaard & Perry Laboratories, Inc., Gaithersburg, MD). Supernatants positive by IFA were screened for blocking of [^35^S]-metabolically labeled EBA-175 erythrocyte binding [4]. Hybridoma cell lines positive by IFA and that blocked native EBA-175 erythrocyte binding to erythrocytes were cloned twice by limiting dilution. Ascites was produced in nude mice (Harlan Bioproducts for Science, Inc., Madison, WI). IgG was purified from ascites by Protein G column chromatography using the ImmunoPure Buffer System (Pierce, Rockford, IL). IgG subclasses were determined by ELISA using a mouse monoclonal antibody isotyping kit (Sigma, St. Louis, MO) as suggested by the manufacturer. The specificity and growth inhibitory activity of the rabbit polyclonal antibodies produced against recombinant EBA-175 RII has been reported [18].

### Immunoprecipitation, immunoblot and erythrocyte blocking of binding studies

Aliquots of approximately 1×10^8^ parasitized (3D7) erythrocytes that had been metabolically labeled for 4 hours were extracted in buffer containing 1% Triton X-100, [36], [37]. Samples were immunoprecipitated by coupling each mAb to Protein G-Sepharose (GE Healthcare) and the immunoprecipitates were washed as previously described using a buffer containing Triton X-100. The labeled proteins were resolved by SDS-PAGE and detected using a phosphoimager (BioRad Molecular Imager FX, Hercules, CA). Immunoblots using the baculovirus recombinant EBA-175 RII protein as well as recombinant F1 and F2 domains expressed in methylotrophic yeast *Pichia pastoris* were prepared as described previously [22]. EBA-175 erythrocyte blocking of binding studies and the determination of the IC_50_ for blocking of EBA-175 binding were performed as previously described, respectively [4], [17].

### Immunofluorescence Assay

Indirect immunofluorescence assays for screening and selection of monoclonal antibody producing clones were performed essentially as described [12] using mouse or rabbit specific antibodies coupled to Alexa 488 or Alexa 546 (Molecular Probes, Eugene, OR), respectively. For other assays including sporozoites, asexual stages including schizonts, male and female gametocytes, retorts and ookinetes and liver stages, parasites were of the NF54 strain. Immunofluorescence assays were performed on 12-well Cel-Line Brand IFAT slides (Erie Scientific), containing air-dried sporozoites, or methanol fixed asexual stages including schizonts, and sexual stage parasites in infected human erythrocytes. For liver stage parasites, slides were incubated in a moist chamber at 37°C for 1 hour with 2 fold diluted (starting from 1∶50) monoclonal antibodies, R217 and 2A10 (mAb against PfCSP) in 1XPBS containing 1% BSA and 0.001% saponin. Slides were washed three times, five minutes each, with 1X PBS and then incubated for 1 h at 37°C with Alexa Fluor 594 anti-mouse antibody at a dilution of 1∶200 in 1XPBS containing 1% BSA and 0.001% saponin and 4', 6-diamidino-2-phenylindole (DAPI, 40 µg/mL) or Alexa Fluor 488 anti-mouse antibody (Green) at a dilution of 1∶200 in 1XPBS containing 1% BSA and 0.001% saponin. After three washes in 1X PBS, slides were mounted with Vectashield mounting medium and observed under phase contrast at X1000 magnification.

### Surface Plasmon Resonance (BIAcore)

The KDs for RII binding by mAbs R217, R218 and R256 were determined using a BIAcore 3000 (BIAcore, Piscataway, NJ). Purified recombinant RII was immobilized on the flow cell of a CM5 BIAcore biosensor chip. The running buffer was HSB-P buffer (10 mM HEPES [pH 7.4], 150 mM NaCl, 0.005% polysorbate 20 adjusted to 1 mM MgCl2) (BIAcore). All measurements were determined at 25°C. The Langmuir method from BIAevaluation 3.0 was used for determination of KDs [38].

### Asexual stage parasite growth inhibition studies

Asexual stage parasite growth inhibition studies were performed as previously described [39]. Briefly, synchronized cultures of *P. falciparum* schizont-infected erythrocytes were mixed with test or control Protein G purified immunoglobulin G (IgG) so that final IgG concentrations were as reported and the hematocrit was 6%. The micro-culture plates were harvested for flow cytometry and stained with Hoechst dye to detect parasite DNA. Percent inhibition was calculated from the mean parasitemias of triplicate test and control wells as 100× (control – test)/control. Polyclonal rabbit anti-EBA-175 RII IgG was used as a positive control for all experiments (data not shown) [18].

### *In vitro* Inhibition of Liver Stage Development Assay (ILSDA)

ILSDA was performed as described previously [40], [41] with some modifications. 4×10^4^ human hepatocyte HCO4 [24] cells in D-MEM/F12 complete medium containing 10% fetal bovine serum were seeded in each well of 8-well LabTek slides. After about 16 hours, medium were aspirated from the wells and 2.5×10^4^ sporozoites (NF54) were mixed with either 100 ug/mL anti-PfCSP mAb 2A10 or 100 ug/mL mAb R217 in 50 uL, and added to each well. Triplicate wells were set up. Three wells of a slide received sporozoites in 50 uL medium only (medium control). Cultures were incubated at 37°C, 5% CO_2_ for 3 hours. Wells were then washed thrice with medium, after which they were replenished with 300 uL media, and returned to incubation at 37°C, 5% CO_2_. Medium was replaced with fresh medium daily. At 72 hours, wells of the slide were washed with 1X PBS thrice and fixed with ice-cold absolute methanol for 10 minutes at room temperature. Fixed slides were washed thrice with 1X PBS. Each well of the slide was incubated with rabbit serum containing polyclonal antibodies against *P. falciparum* liver stage antigen-1 (PfLSA -1) at 1∶50 dilution followed by Alexa Fluor 488 anti-rabbit secondary antibody. Slides were mounted with Vectashield and viewed using an epifluorescent microscope. The numbers of parasites expressing PfLSA-1 (p) present in each well were counted and percent inhibition was calculated as follows:
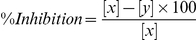
where *x*  =  Mean number of (p) in medium control wells

where *y*  =  Mean number of (p) in Test mAb wells

### Expression of EBA-175 in hepatic stages of *P. falciparum* in human hepatocytes *in vitro*

#### Early hepatic stage (3 day) assay

LabTek chamber slide wells were seeded with 4×10^4^ HC-O4 cells. After twenty four hours, 25,000 sporozoites were added to each well. Washing and processing of slides were as described in the ILSDA above. After 3 days, wells were fixed and stained with either rabbit polyclonal antiserum against *P. falciparum* LSA-1 (1∶50 dilution) followed by Alexa Fluor 488 anti-rabbit antibody or R217 (100 ug/mL), followed by Alexa Fluor 488 anti-mouse antibody and assessed as mentioned above. The numbers of parasites expressing PfLSA-1 or PfEBA-175 in each well were counted.

#### Late hepatic stage (6 day) assay

LabTek chamber slide wells were seeded with 4×10^4^ HC-O4 cells. After twenty four hours, 50,000 sporozoites were added to each well, and processed and maintained as above. After 6 days, wells were fixed and stained with either rabbit antiserum against *P. falciparum* LSA-1 (1∶50 dilution) followed by Alexa Fluor 488 anti-rabbit antibody or mAb R217 (100 ug/mL), followed by Alexa Fluor 488 anti-mouse antibody and assessed as mentioned above. The numbers of parasites expressing PfLSA-1 or PfEBA-175 in each well were counted.
